# Parasitological and malacological surveys to identify transmission sites for *Schistosoma mansoni* in Gomma District, south-western Ethiopia

**DOI:** 10.1038/s41598-022-21641-2

**Published:** 2022-10-12

**Authors:** Teshome Bekana, Endegena Abebe, Zeleke Mekonnen, Begna Tulu, Keerati Ponpetch, Song Liang, Berhanu Erko

**Affiliations:** 1 Department of Medical Laboratory Sciences, College Health Sciences, Mettu University, Mettu, Ethiopia; 2 Aklilu Lemma Institute of Pathobiology, Addis Ababa University, Addis Ababa, Ethiopia; 3 Department of Biomedical Sciences, College Health Sciences, Mettu University, Mettu, Ethiopia; 4 Department of Medical Laboratory Sciences, Institute of Health, Jimma University, Jimma, Ethiopia; 5 Department of Medical Laboratory Sciences, Bahir Dar University, Bahir Dar, Ethiopia; 6 Faculty of Public Health and Allied Health Sciences, Ministry of Public Health, Sirindhorn College of Public Health Trang, Praboromarajchanok Institute, Nonthaburi, Thailand; 7 Department of Environmental and Global Health, College of Public Health and Health Professions, Emerging Pathogens Institute, University of Florida, Gainesville, FL 32610 USA

**Keywords:** Parasitology, Microbiology, Medical research

## Abstract

Schistosomiasis is a neglected tropical disease that disproportionately affects the poorest people in tropical and subtropical countries. It is a major parasitic disease causing considerable morbidity in Ethiopia. Despite significant control efforts, schistosomiasis transmission is still widespread in many rural areas of the country. The aim of this study was to determine the prevalence and intensity of intestinal schistosomiasis among schoolchildren, as well as to identify schistosomiasis transmission sites in Gomma District, southwestern Ethiopia. Between October 2018 and September 2019, cross-sectional parasitological and malacological surveys were conducted in the study area. The study comprised 492 school-children aged 6 to 15 years old from four primary schools in Gomma District. To identify and quantify eggs of *Schistosoma mansoni* from the children, stool specimens were collected and processed using double Kato-Katz thick smears. Water bodies adjacent to human settlements in the study area were surveyed for snail intermediate hosts of *S. mansoni.* Morphological identification of collected snails was conducted, followed by examining their infection status using a dissecting microscope. The overall prevalence of *S. mansoni* infection was 73.8% (95%CI: 69.9–77.7%) and 41.6% of them had moderate-to-heavy infections. The prevalence of *S. mansoni* infection differed considerably by age group, with the older age groups (12–15) having a higher prevalence than the younger age groups (6–11) (p < 0.001). The prevalence of infection also varied significantly among schools; Dedo Ureche had the highest prevalence (86.9%) (p = 0.034), while Goga Kilole had relatively the lowest prevalence of *S. mansoni* infection (59.6%) (p = 0.003). A total of 1463 *Biomphalaria pfeifferi* snails were collected from 11 survey sites throughout the study area, with 357 (24.4%) of the snails shedding schistosomes cercariae. Despite intensified efforts to scale up mass drug administration in Ethiopia, this study reported high levels of *S. mansoni* infection among schoolchildren and snail intermediate hosts in rural communities in Gomma. Such a high infection rate warrants pressing needs for targeted and integrated interventions to control the disease in the area.

## Introduction

Schistosomiasis is a water-based debilitating disease caused by parasitic trematodes of the genus *Schistosoma*^1^*.* It is endemic in more than 70 countries and affects around 240 million people globally, with an estimated global burden of 1.9 million disability-adjusted life years (DALYs) in 2016^2,3^. Schistosomiasis is one of the many neglected tropical diseases (NTDs) that mostly affect people in sub-Saharan Africa. The disease is caused by human *schistosoma* species, which are transmitted via contact with freshwater bodies contaminated with cercariae, the free-swimming larval stage of schistosomes released by the intermediate host snail^4^.

*Schistosoma mansoni*, which causes intestinal schistosomiasis, and *S. haematobium*, which causes urogenital schistosomiasis, are the two schistosome species that infect humans in Ethiopia^5^. The former has been widely documented in various parts of the country, while the latter is restricted to a few lowland locations^5,6^*.* Freshwater snails *Biomphalaria pfeifferi* and *B*. *sudanica* transmit and maintain intestinal schistosomiasis, while *Bulinus* abyssinicus and *Bu*. *africanus* are the principal intermediate hosts for urogenital schistosomiasis transmission in Ethiopia^7^. The disease is a major health problem in the country, particularly in rural communities with poor access to clean water supplies and adequate sanitation. It is estimated that about 5 million people are infected, and more than 37 million people live in areas with high risk of schistosomiasis transmission in the country^5^. Despite the lack of recent estimates of the disease's burden in Ethiopia, parasitological surveys conducted in several parts of the country have shown that the prevalence of schistosomiasis is on the increase, particularly in connection with intense population movement and water resource development^8,9^.

Ethiopia has been implementing school-based mass drug administration by periodically giving praziquantel to schoolchildren in areas with a moderate to high prevalence of schistosomiasis since the start of the national preventive chemotherapy program in 2015^10^. According to a Federal Ministry of Health (FMoH) report, praziquantel was administered to more than 19 million schoolchildren between 2015 and 2019^11^. However, despite substantial efforts to scale up mass deworming programs in various parts of the country, treatment coverage in some rural areas has yet to be achieved, partly due to a lack of verified schistosomiasis transmission. In the present study area, factors that favor the transmission of schistosomiasis include the presence of several water bodies used for domestic activities, a lack of safe water supply, and the occurrence of the snail intermediate host. In this context, epidemiological surveys are required to identify the transmission foci of schistosomiasis and design effective interventions. Therefore, the aims of this study were to determine the prevalence and intensity of *S. mansoni* infection among schoolchildren and to identify transmission sites for schistosomiasis in Gomma District, southwestern Ethiopia.

## Materials and methods

### Study setting

**Figure 1:**
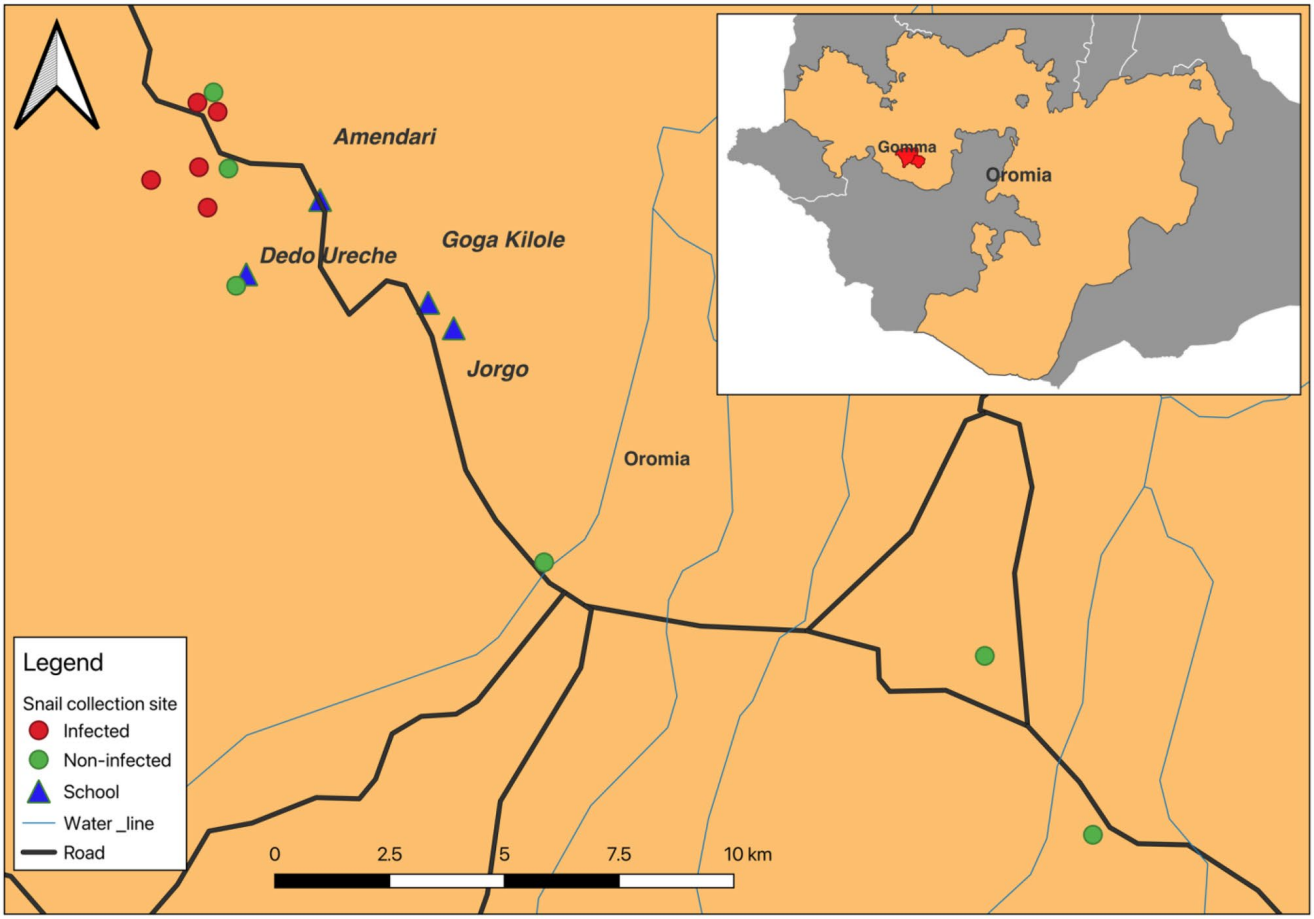
Map of study area.

The study was conducted in Gomma, a district situated in Jimma Zone, southwestern Ethiopia (Fig. 1). The climatic condition of the area is characterized by a short rainy season from April to May, a long rainy season from June to September, and a dry season from January to March. The area has an elevation of 1563–1732 m above sea level with a daily mean temperature ranging from 13.4 to 27 °C. The people in the area are subsistence farmers who largely rely on animal husbandry and agriculture for their livelihoods. Food crops and fruit trees planted in the areas are maize (*Zea mays*), rice, beans, teff, mango, avocado, and citrus fruits. Several water bodies, including perennial rivers, small streams, canals, and rain-filled ponds are located in the study area. These water bodies are primarily used for drinking, laundering, swimming and other recreational and domestic activities.

### Study design and sample size calculation

A cross-sectional study involving parasitological and malacological surveys was conducted between October 2018 and September 2019 in the study area. The sample size was calculated using a single proportion formula $\mathrm{n}=\frac{{\mathrm{Z}}^{2}\left(1-\frac{\mathrm{\alpha }}{2}\right)\mathrm{P}(1-\mathrm{P})}{{\mathrm{d}}^{2}}$, where n is the sample size, Z is the statistics corresponding to a 95% level of confidence (1.96), d is the precision of the estimate, and P is the assumed prevalence of intestinal schistosomiasis (50%). The infection prevalence was assumed to be 50% because the current status of *S.mansoni* infection in the area is unknown. The sample size was adjusted to 492 by including an additional 115 (30%) children to compensate for the refusal to submit stool specimens and to reduce errors that may occur during Kato-Katz preparation. Accordingly, 121 children from Amendari, 123 from Dedo Ureche, 119 from Goga Kilole, and 129 from Jorgo primary school participated in the study.

### Study population, recruitment and sampling techniques

Schoolchildren aged 6–15 years who lived in the study area for three months or more and who were attending the selected schools at the time of data collection were considered as the study population. Four primary schools, namely Amendari, Dedo Ureche, Goga Kilole, and Jorgo, were purposely selected based on their proximity to water bodies (rivers and/or streams) in the area. Before the actual data collection, the number of children aged 6–15 years old who were attending grade 1–5 was obtained from school principals at all schools. A quota was then allocated to each school with proportional allocation according to the number of eligible children in each school. Participating children were selected by a systematic random sampling technique using students’ enrollment list as a sampling frame.

### Parasitological examination

Stool specimens were collected from schoolchildren in December 2018 after their parents or guardians provided written informed consent. In brief, each child was given a clean plastic sheet with a wooden applicator stick to bring about a 2 g stool specimen. Two Kato-Katz smears were prepared from each stool specimen using a 41.7 mg template. The Kato-Katz thick smears were prepared at schools and then transported to the laboratory for microscopic examination at the Aklilu Lemma Institute of Pathobiology at Addis Ababa University. The prepared smears were microscopically examined to identify and quantify eggs of *S. mansoni*. To estimate the intensity of infections, eggs counted in both Kato-Katz thick smears were multiplied by a factor of 24 to obtain the faecal egg count in units of eggs per gram of stool (EPG). According to World Health Organization (WHO)^12^, the intensity of *S. mansoni* infection was classified as light (1–99 EPG), moderate (100–399 EPG), and heavy (> 400 EPG).To ensure consistency in egg counting, 10% of the examined Kato-Katz smears were re-examined by a senior laboratory technician.

### Ethical consideration

Ethical clearance was obtained from the Institutional Review Board (IRB) of the Aklilu Lemma Institute of Pathobiology, Addis Ababa University (Ref.:ALPB/IRB/027/2012/2019) and all methods were performed in accordance with relevant guidelines and regulations. Permission for the study was also obtained from the District Health and Educational Bureaus. School teachers, parents/guardians, and children were informed about the purposes, procedures, and usefulness of the study. Only children who were willing to participate in the study and whose parents or legal guardians signed the informed consent forms were allowed to participate in the study. Children infected with *S. mansoni* were treated with a single 40 mg/kg oral dose of praziquantel in accordance with WHO guidelines^12^. All children found positive for other intestinal helminths were treated with appropriate drugs.

### Snail survey

Snail survey was conducted at 11 water bodies (Agaro, Buluqute, Gembe, Jawe, Naggo Hagayyo, Qarqir, Semma, Yachi, Burqa, Yamo, and Yisa), which included seasonal streams, ponds, a dam, and swamps (Table S1). The snails were collected by two field collectors from water bodies containing submerged vegetation, decaying woods and leaves using a wire mesh scoop attached to a wooden handle for the duration of 15 min at each collection site. A snail survey was conducted after the short rainy season (October to December 2018), during the dry season (January to March 2019) and during the rainy season (June to September 2019). The collected snails were transported to the Medical Laboratory of the Aklilu Lemma Institute of Pathobiology in pre-labelled plastic containers containing aquatic weeds and pond water. The snails were then identified to the genus and species level based on shell morphology using the key to the identification of freshwater snails^13^. The snails were placed individually in shedding vials containing aged water and exposed to electric light for a duration of 30 min to an hour. Snails that were not positive on the first exposure were re-checked on the second day for cercariae shedding. Cercariae shed from the snails were identified using the identification key of Frandsen and Christensen (1984)^14^.

### Data analysis

Data were entered using Microsoft Excel version 2007 (MS Corporation, Washington, USA), cleaned for error and analysis was carried out using the Statistical Package for Social Scientists (SPSS) version 22. (SPSS Inc, Chicago, IL, USA). Descriptive statistics were employed to compute the prevalence and mean intensity of *S. mansoni* infections by age group, sex, and schools. Logistic regression analysis was used to identify factors associated with *S. mansoni* infection. A Student's t-test or ANOVA were used to compare the arithmetic mean intensity of infections. Odds ratios and 95% confidence intervals were calculated. Associations or differences with P values of less than 5% were considered statistically significant.

## Results

### Demographic characteristics of the study participants

A total of 492 schoolchildren were included in the study. Of these, 289 (58.7%) were boys and 203 (41.3%) were girls. Their age ranged from 6 to 15 years old with a mean age of 10.9 (standard deviation (SD) = 2.1 years). The study participants were selected from four primary schools: 121 (24.6%), 123 (25%), 119 (24.2%), and 129 (26.2%) from Amendari, Dedo Ureche, Goga Kilole, and Jorgo primary schools, respectively.

### Prevalence of *S. mansoni* infection

The prevalence of *S. mansoni* infection according to age, gender, and school is summarized in Table 1. Of the total 492 study participants, 73.8% (95% CI: 69.9–77.7%) were positive for *S. mansoni* eggs. The prevalence of *S.mansoni* infection was significantly higher among children aged 12–15 years (85.5%, 95% CI: 81.1–89.8%) compared to those aged 9–11 years (62.7%, 95% CI: 54.9–70.1%) and 6–8 years (60.6%, 95% CI: 50.7–70.1%). The prevalence of infection varied by schools; Dedo Ureche had the highest prevalence of infection (86.9% ), followed by Amendari (80.9%), Jorgo (67.4%), and Goga Kilole primary school (59.6%). However, the difference in the prevalence of *S. mansoni* infection was not significantly different between males and females (p = 0.708) (Table 1). Other intestinal helminth parasites encountered in this study were *Trichuris trichiura* (23.2%), *Ascaris lumbricoides* (8.5%), *Enterobius vermicularis* (3.6%), and *Hymenolepis nana* (3.0%).

**Table 1:** Prevalence and mean intensity of *S.mansoni* infection among the school children in Gomma District, Southwestern Ethiopia.

Variable	N	Prevalence (%) (95% CI)	Univariate COR (95% CI)	Multivariate AOR (95% CI)	Intensity of Infection			
					Light infection n (%)	Moderate infection n (%)	Heavy infection n (%)	AM (95% CI)
Overall	492	73.8 (69.9–77.7)			158 (32.1)	101(20.5)	104 (21.1)	446.1 (379.5–512.6)
**Age (years)**								
6–8	94	60.6 (50.7–70.5)	0.3 (0.2–0.4)	0.2 (0.1–0.4)**	23 (24.5)	19 (20.2)	15 (15.9)	738.1 (476.4–999.8)
9–11	150	62.7 (54.9–70.1)	0.3 (0.2–0.5)	0.2 (0.1–0.4)**	42 (28.0)	23 (15.3)	29 (19.3)	442.7 (337.5–548.0)
12–15	248	85.5 (81.1–89.8)	1	1	93 (37.5)	59 (23.8)	60 (24.2)	369.0(299.7–438.3)
**Gender**								
Male	289	75.1 (70.1–80.1)	0.8 (0.6–1.3)	0.9 (0.6–1.4)	90 (31.1)	60 (20.7)	67 (23.2)	448.7 (299.4–597.9)
Female	203	71.9 (65.7–78.1)	1	1	68 (33.5)	41 (20.2)	37 (18.2)	437.6 (331.9–543.3)
**Schools**								
Amendari	121	80.9 (73.9–87.9)	2.1 (1.1–3.7)	1.8 (0.9–3.4)	41 (33.8)	35 (28.9)	22 (18.2)	456.5 (295.3–617.7)
Dedo Ureche	123	86.9 (81.0–92.9)	3.2 (1.7–6.1)	2.1 (1.1–4.1)*	49 (39.8)	21 (17.1)	37 (30.1)	50.9 (363.7–656.2)
Goga Kilole	119	59.6 (50.8–68.5)	0.7 (0.4–1.2)	0.4 (0.2–0.7)*	33 (27.7)	25 (21.0)	13 (10.9)	247.1 (190.5–303.7)
Jorgo	129	67.4 (59.3–75.5)	1	1	35 (27.1)	20 (15.5)	32 (24.8)	518.1 (396.1–640.1)

*AM* arithmetic mean, *AOR* adjusted odds ratio, *CI* confidence interval, *COR* crude odds ratio, *N* number examined.

*Statistical significance at P < 0.05.

**Statistical significance at P < 0.001.

### Intensity of *S. mansoni* infection

Table 1 shows the intensity of *S. mansoni* infection by age, gender, and school. The overall arithmetic mean of *S. mansoni* eggs per gram of stool was 446.1 (ranging from 24 to 4800 EPG). Out of 363 children infected with *S. mansoni*, 32.1%, 20.5%, and 21.1% had light, moderate, and heavy infections, respectively. There was no significant difference between male and female children in infection intensity (t = 0.395, p = 0.971). According to age group, the highest infection intensity was observed in the 6–8 year age group (738.1 EPG, 95% CI: 476.4–999.8), followed by 9–11 years (442.7 EPG, 95% CI: 337.5–548.0) and 12–15 years (369.0 EPG, 95% CI: 299.7–438.3). However, the infection intensity was not significantly varied according to age (F (2,489) = 1.938, p = 0.145).

### Snail survey

A total of 1981 freshwater snails were collected from 11 human water contact sites over the course of the study. Based on morphological characteristics, 1463 (73.8%) of the snails were putatively identified as *Biomphalaria pfeifferi* and 518 (26.1%) as *Lymnaea natalensis. B. pfeifferi* was found at eight collection sites, while *Lymnaea natalensis* was found at five sites. The highest number of *B. pfeifferi* was recorded in Yisa (412 snails), followed by Buluqute (274 snails) and Yamo (252 snails) (Table 2).

**Table 2:** The proportion of infected *B. pfeifferi* by collection sites and seasons.

**Sites**	Number of *B. pfeifferi* collected (% of infection)			
	Post-rainy season	Dry season	Rainy season	Total
Agaro	0	0	0	0
Buluqute	179 (35.7)	95 (24.2)	0	274 (31.7)
Gembe	64 (0)	15 (0)	3 (0)	82 (0)
Jawe	0	0	0	0
Naggo Hagayyo	78 (2.6)	34 (0)	0	112 (1.8)
Qarqir	207 (85.5)	9 (66.7)	0	216 (85.2)
Semma	15 (0)	6 (0)	0	21 (0)
Yachi	0	0	0	0
Burqa	68	26	0	94
Yamo	169 (14.2)	83 (14.4)	0	252 (14.3)
Yisa	297 (15.1)	90 (3.3)	25 (0)	412 (11.6)
Total	1077 (28.9)	358 (12.3)	28 (0)	1463 (24.4)

Out of the *B. pfeifferi* examined using a dissecting microscope, 24.4% (357/1463) were infected with schistosomes. The proportion of cercariae-shedding *B. pfeifferi* snails in the collection sites ranged from (2/112) 1.8% (Naggo Hagayyo) to 184/216 (85.2%) (Qarqir). Overall, 5 collection sites had *B. pfeifferi* infected with schistosomes (Fig. 1). At two collection sites (Qarqir and Buluqute), over 30% of the examined snails were infected. At Naggo Hagayyo, Yamo, and Yisa collection sites, the prevalence of infected snails was below 15%, while at the other three collection points (Agaro, Jawe, and Yachi) no infected snails were observed. According to season, the highest *B. pfeifferi* infection rate was observed post-rainy season (October to December 2018) (28.9%) followed by the dry season (January to March 2019) (12.3%), while no infected snail was observed during the rainy season (June to September 2019) (Table 2).

## Discussion

Epidemiological data on schistosomiasis prevalence in human and snail intermediate hosts is essential for identifying transmission sites, determining the disease's distribution in a given area, and informing control programs. In this study, 73.8% of the participants had intestinal schistosomiasis. The high prevalence of intestinal schistosomiasis observed was consistent with findings reported from Bushulo village, southern Ethiopia (73.7%)^15^, Demba Girara, southern Ethiopia (81.3%)^16^ and Sanja town, northern Ethiopia (82.8%)^17^. In contrast, the prevalence of *S*. *mansoni* infection observed in the present study was higher than previous studies from Ethiopia (2.1%-29%)^18–20^, Kenya (12.2%)^21^, Nigeria (12.1%)^22^, Tanzania (10.7%)^23^ and from Uganda (10.7%)^24^. The high prevalence of *S. mansoni* infection in our setting might be attributable to inadequate sanitary facilities^25^, unsafe water supply, absence of effective control measures and the availability of several water sources used for bathing, swimming and other domestic and recreational purposes.

The prevalence of *S.mansoni* infection was significantly higher among children aged 12–15 years compared to the lower age groups (6–11 years), which is consistent with studies carried out in Ethiopia^26^, Zambia^27^, Yemen^28^, Senegal^29^, South Africa^30^, Nigeria^31^ and Sudan^32^. Children in higher age groups are at a higher risk of infection, likely due to increased engagement in water-contact activities such as swimming and watering animals, whilst those in lower age groups have limited exposure to ponds or other bodies of water. This could also be due to the fact that when children get older, they are more likely to spend their time around cercariae-infested water, resulting in a higher accumulated high risk of acquiring schistosomes. This finding, however, contradicts research from Brazil^33^, Ghana^34^, and Kenya^35^, which found that *S. mansoni* infection was more common in younger children. The variation could be attributable to differences in water contact frequency and duration, environmental exposure, or social and cultural practices.

The prevalence of moderate-intensity plus heavy-intensity infection among the infected schoolchildren in the present study was 41.67%. This is considerably higher compared to the goals set by WHO (reducing the prevalence of heavy-intensity infection to 1% in all endemic areas by 2025) for achieving the elimination of schistosomiasis as a public health problem^12,36,37^. The high infection intensity found in this study shows that the study area has a high schistosomes exposure and transmission rate. This was confirmed by collecting schistosomes infected snails from the collection sites, indicating the area as a local transmission hotspot for schistosomiasis. Therefore, children and other high-risk groups living in the study area should be treated through the large-scale administration of praziquantel to reduce morbidity and interrupt disease transmission.

The results of this study also revealed that children attending Dedo Ureche Primary School had a higher prevalence of *S. mansoni* infection than children attending other schools. This was likely due to the presence nearby freshwater source (Qarqir stream) infested with schistosomes infected snails, where the children could contract the infection while playing, bathing, and engaging in other activities like washing clothes and fetching water.

Intermediate host snails play a crucial role in locating hotspot areas for transmission of schistosomiasis, planning intervention, and evaluating the efficacy of ongoing control strategies. The present malacological survey shows that most of the collection sites in the study area had high *B. pfeifferi* abundance and about one-fourth of the snails (24.4%) were shedding cercariae of *S. mansoni*. Our findings on the high prevalence of infected snails are consistent with the findings of a recent malacological survey in Ethiopia, which found high snail abundance and the presence of infected *B. pfeifferi* (30.5%) along the shorelines of Ethiopian Rift Valley lakes^38^. In addition, a recent review of schistosomiasis-infected snails found that 15.9% of *Biomphalaria* snails shed schistosomes cercariae in Ethiopia^9^. The high prevalence and intensity of *S. mansoni* infection among schoolchildren, as well as the recovery of infected intermediate host snails in water bodies, suggests that the area should be considered for preventive chemotherapy, snail control and improved sanitation coupled with regular provision of health information on the risk factors to control the disease.

The number of *B. pfeifferi* snails collected in the present survey varied among the time periods (Table 2). This observation is consistent with the findings of earlier studies that have associated variation in snail distribution with study time. Contrasting results have been reported from Tanzania^39^, Kenya^40^ and Niger^41^, indicating that schistosomiasis-infected snails were high during a dry season. The difference in ecology and rain fall pattern was likely to contribute to the difference observed between the study areas. Most of the identified water sources for snail collection in the present study area were temporary streams or ponds, which dry up throughout the lengthy dry season (January to May), resulting in a low quantity of snails retrieved at this time. When the rains come, however, the dried water bodies begin to re-fill, creating suitable conditions for snails to trigger out of aestivation and repopulate the habitats. Some conditions in bodies of water, including the presence or absence of submerged aquatic hydrophytes, biological factors, and physical and chemical characteristics of the water, could also play a role in snail density variation.

The present study has some limitations that should be noted when interpreting the data. First, despite the fact that using a double Kato-Katz thick smear increases the amount of stool specimens examined, each child only submitted one stool specimen. However, because there can be significant day-to-day variation in egg output in infected children and uneven distributions of eggs in stool specimens, the prevalence and number of eggs recorded might have been underestimated. Second, we used the cercariae shedding technique to determine the infection status of snails, but this method has its own limitations in terms of detecting infection in situations like low parasite burden, sporocyst aborted development, and early prepatent stages. This might have also underestimated the true prevalence of infection in the snail intermediate hosts.

## Conclusions

The study demonstrated the area as a high-risk zone for schistosomiasis because of the high prevalence and intensity of *S. mansoni* infection among the children. The study also revealed that nearly one-fourth of the *Biomphalaria* snails sampled shed schistosomes cercariae, indicating that the location is a schistosomes transmission hotspot. Therefore, repeated administration of praziquantel to schoolchildren along with integrated interventions focusing on health information, snail control, improved sanitation, and provision of safe water supplies are essential to interrupt the transmission and eliminate the disease as a public health problem in the study area.

## Supplementary Information

Supplementary Table S1.

## Data Availability

The dataset analysed during the current study are available from the corresponding author on reasonable request.

## References

[1] Colley DG, Bustinduy AL, Secor WE, King CH (2014). Human schistosomiasis. Lancet.

[2] GBD 2016 DALYs and HALE Collaborators. Global, regional, and national disability-adjusted life-years (DALYs) for 333 diseases and injuries and healthy life expectancy (HALE) for 195 countries and territories, 1990–2016: A systematic analysis for the Global Burden of Disease Study 2016. *Lancet* **390**, 1260–1344 (2017). 10.1016/S0140-6736(17)32130-X PMC5605707 28919118

[3] Barry MA, Simon GG, Mistry N, Hotez PJ (2013). Global trends in neglected tropical disease control and elimination: Impact on child health. Arch. Dis. Childh. Lond..

[4] Hotez PJ (2014). The Global Burden of Disease Study 2010: Interpretation and implications for the neglected tropical diseases. PLoS Negl. Trop. Dis..

[5] Deribe K (2012). The burden of neglected tropical diseases in Ethiopia, and opportunities for integrated control and elimination. Parasites Vectors..

[6] Jember TH (2014). Challenges of schistosomiasis prevention and control in Ethiopia: Literature review and current status. J. Parasitol. Vector Biol..

[7] Kloos H (1988). Schistosomiasis in Ethiopia. Soc. Sci. Med..

[8] Chala B, Torben W (2018). An epidemiological trend of urogenital schistosomiasis in Ethiopia. Public Health Front..

[9] Hailegebriel T, Nibret E, Munshea A (2021). Prevalence of *Schistosoma **mansoni* and associated risk factors in human and biomphalaria snails in Ethiopia: A systematic review and meta-analysis. Acta Parasitol..

[10] World Health Organization. *Schistosomiasis: Progress Report 2001–2011, Strategic Plan 2012–2020*. (WHO, 2013).

[11] Leta GT (2020). National mapping of soil-transmitted helminth and schistosome infections in Ethiopia. Parasites Vectors..

[12] World Health Organization. *Prevention and Control of Schistosomiasis and Soil-Transmitted Helminthiasis: Report of a WHO Expert Committee*. (WHO, 2002). 12592987

[13] Brown DS (1984). Freshwater Snails of Africa and Their Medical Importance.

[14] Frandsen F, Christensen N (1984). An introductory guide to the identification of cercariae from African freshwater snails with special reference to cercariae of trematode species of medical and veterinary importance. Acta Trop..

[15] Terefe A, Shimelis T, Mengistu M, Hailu A, Erko B (2011). Schistosomiasis mansoni and soil-transmitted helminthiasis in Bushulo village, southern Ethiopia. Ethiop. J. Health Dev..

[16] Alemayehu B, Tomass Z (2015). Schistosoma mansoni infection prevalence and associated risk factors among schoolchildren in Demba Girara, Damot Woide District of Wolaita Zone, Southern Ethiopia. Asian Pac. J. Trop. Med..

[17] Alebie G, Erko B, Aemero M, Petros B (2014). Epidemiological study on Schistosoma mansoni infection in Sanja area, Amhara region, Ethiopia. Parasites Vectors.

[18] Ghiwot Y, Degarege A, Erko B (2014). Prevalence of intestinal parasitic infections among children under five years of age with emphasis on *Schistosoma **mansoni* in Wonji Shoa Sugar Estate, Ethiopia. PLoS ONE.

[19] Gebreyesus TD (2020). Prevalence, intensity, and correlates of schistosomiasis and soil-transmitted helminth infections after five rounds of preventive chemotherapy among school children in Southern Ethiopia. Pathogens..

[20] Yami A, Mamo Y, Kebede S (2011). Prevalence and predictors of intestinal helminthiasis among school children in Jimma zone; a cross-sectional study. Ethiop. J. Health Sci..

[21] Amollo D, Kihara J, Kombe Y, Karanja S (2013). Prevalence and intensity of single and mixed schistosoma mansoni and schistosoma haematobium infections in primary school children in Rachuonyo North District, Homabay County, Western Kenya. East Afr. Med. J..

[22] Banji B, Babadoko M, Mohammed G (2012). Survey of schistosomiasis and other intestinal helminthiases among school-aged children in Agaie, Niger state, Nigeria. J. Pharm. Biomed. Sci..

[23] Mazigo HD, Kirway L, Ambrose EA (2019). Prevalence and intensity of *Schistosoma **mansoni* infection in pediatric populations on antiretroviral therapy in north-western Tanzania: A cross-sectional study. BMJ Open.

[24] Stanton MC (2017). Intestinal schistosomiasis in Uganda at high altitude (> 1400 m): Malacological and epidemiological surveys on Mount Elgon and in Fort Portal crater lakes reveal extra preventive chemotherapy needs. Infect. Dis. Poverty..

[25] Beyene A, Hailu T, Faris K, Kloos H (2015). Current state and trends of access to sanitation in Ethiopia and the need to revise indicators to monitor progress in the Post-2015 era. BMC Public Health.

[26] Essa T, Birhane Y, Endris M, Moges A, Moges F (2013). Current status of *Schistosoma **mansoni* infections and associated risk factors among students in Gorgora town, Northwest Ethiopia. Int. Sch. Res. Notices..

[27] Agnew-Blais J (2010). Schistosomiasis haematobium prevalence and risk factors in a school-age population of peri-urban Lusaka, Zambia. J. Trop. Pediatr..

[28] Sady H (2013). Prevalence and associated factors of schistosomiasis among children in Yemen: implications for an effective control programme. PLoS Negl. Trop. Dis..

[29] Senghor B (2014). Prevalence and intensity of urinary schistosomiasis among school children in the district of Niakhar, region of Fatick, Senegal. Parasites Vectors.

[30] Kabuyaya M, Chimbari MJ, Mukaratirwa S (2018). Infection status and risk factors associated with urinary schistosomiasis among school-going children in the Ndumo area of uMkhanyakude district in KwaZulu-Natal, South Africa two years post-treatment. Int. J. Infect. Dis..

[31] Oluwole AS (2018). Prevalence, intensity and spatial co-distribution of schistosomiasis and soil transmitted helminths infections in Ogun state, Nigeria. Parasitol. Open..

[32] Tamomh A, Yousfi S, Abakar A, Nour B (2018). Prevalence of intestinal Schistosomiasis among basic school children in White Nile sugar scheme a new irrigated project, White Nile state, Sudan. Biol. Med. (Aligarh).

[33] Enk MJ (2010). Factors related to transmission of and infection with *Schistosoma **mansoni* in a village in the South-eastern Region of Brazil. Mem. Inst. Oswaldo Cruz..

[34] Nkegbe E (2010). Sex prevalence of schistosomiasis among school children in five communities in the lower river Volta basin of South Eastern Ghana. Afr. J. Biomed. Res..

[35] Odiere MR (2012). High prevalence of schistosomiasis in Mbita and its adjacent islands of Lake Victoria, western Kenya. Parasites Vectors.

[36] Montresor A, Engels D, Ramsan M, Foum A, Savioli L (2002). Field test of the ‘dose pole’for praziquantel in Zanzibar. Trans. R. Soc. Trop. Med. Hyg..

[37] World Health Organization. *Ending the Neglect to Attain the Sustainable Development Goals: A Road Map for Neglected Tropical Diseases 2021–2030*. (WHO, 2020).

[38] Olkeba BK (2021). Malacological and parasitological surveys on Ethiopian rift valley lakes: Implications for control and elimination of snail-borne diseases. Int. J. Environ. Res. Public Health..

[39] Gouvras AN (2017). Longitudinal survey on the distribution of *Biomphalaria*
*sudanica* and *B.*
*choanomophala* in Mwanza region, on the shores of Lake Victoria, Tanzania: Implications for schistosomiasis transmission and control. Parasites Vectors..

[40] Muhoho ND (1997). Cercarial density in the river of an endemic area of schistosomiasis haematobia in Kenya. Am. J. Trop. Med..

[41] Rabone M (2019). Freshwater snails of biomedical importance in the Niger River Valley: Evidence of temporal and spatial patterns in abundance, distribution and infection with Schistosoma spp. Parasites Vectors..

