# Supplementary material for: Parasitological and malacological surveys to identify transmission sites for Schistosoma mansoni in Gomma District, south-western Ethiopia
Source: Sci Rep. 2022 Oct 12;12:17063. doi: 10.1038/s41598-022-21641-2 (PMC9556602; doi:10.1038/s41598-022-21641-2)
Supplement: Supplementary file 1 — Supplementary Table S1. [file 41598_2022_21641_MOESM1_ESM.pdf]

**Parasitological and malacological surveys to identify transmission sites for *Schistosoma mansoni* in Gomma District, south-western Ethiopia**

Teshome Bekana*[1, 2], Endegena Abebe[3], Zeleke Mekonnen[4], Begna Tulu [5], Keerati Ponpetch [6], Song Liang[7], Berhanu Erko[2]

**Table S1**.Sampling sites and infection status of snails in Gomma District, showing the types of water bodies, the coordinates of snail sampling points and the season sampled

| **Sampling date** | Month/Season | Site (types of water bodies) | Coordinates | Number of B. pfeifferi collected | Number of B. pfeifferi shedding cercariae |
|---|---|---|---|---|---|
| 14/10/2018 | After rainy season | Agaro (stream) | N-7.865111, E-36.574813 | 0 | 0 |
| 14/10/2018 | After rainy season | Buluqute (stream) | N-7.955925, E-36.506322 | 179 | 64 |
| 18/10/2018 | After rainy season | Gembe(stream) | N-7.846611,E-36.661874 | 64 | 0 |
| 18/10/2018 | After rainy season | Jawe (river) | N-7.957959, E-36.509508 | 0 | 0 |
| 18/10/2018 | After rainy season | Naggo Hagayyo (Swamp) | N-7.954103, E-36.510323 | 78 | 2 |
| 18/10/2018 | After rainy season | Qarqir(stream) | N-7.93517, E-36.508335 | 207 | 177 |
| 21/10/2018 | After rainy season | Semma (river) | N-7.91972, E-36.513993 | 15 | 0 |
| 1/11/2018 | After rainy season | Yachi (Irrigation furrow) | N-7.942895, E-36.512464 | 0 | 0 |
| 1/11/2018 | After rainy season | Burqa(spring) | N-7.811247, E-36.683232 | 68 | 0 |
| 1/11/2018 | After rainy season | Yamo(dam) | N-7.940623, E-36.497171 | 169 | 24 |
| 1/11/2018 | After rainy season | Yisa(stream) | N-7.943158, E-36.506631 | 297 | 45 |
| 12/1/2019 | Dry Season | Agaro (stream) | N-7.865111, E-36.574813 | 0 | 0 |
| 12/1/2019 | Dry Season | Buluqute (stream) | N-7.955925, E-36.506322 | 95 | 23 |
| 12/1/2019 | Dry Season | Gembe(stream) | N-7.846611,E-36.661874 | 15 | 0 |
| 12/1/2019 | Dry Season | Jawe (river) | N-7.957959, E-36.509508 | 0 | 0 |
| 1/5/2019 | Dry Season | Naggo Hagayyo (Swamp) | N-7.954103, E-36.510323 | 34 | 0 |
| 1/5/2019 | Dry Season | Qarqir(stream) | N-7.93517, E-36.508335 | 9 | 7 |
| 1/5/2019 | Dry Season | Semma (river) | N-7.91972, E-36.513993 | 6 | 0 |
| 1/5/2019 | Dry Season | Yachi (Irrigation furrow) | N-7.942895, E-36.512464 | 0 | 0 |
| 1/5/2019 | Dry Season | Burqa(spring) | N-7.811247, E-36.683232 | 26 | 0 |
| 1/5/2019 | Dry Season | Yamo(dam) | N-7.940623, E-36.497171 | 83 | 12 |
| 1/5/2019 | Dry Season | Yisa(stream) | N-7.943158, E-36.506631 | 90 | 3 |
| 19/6/2019 | Rainy season | Agaro (stream) | N-7.865111, E-36.574813 | 0 | 0 |
| 19/6/2019 | Rainy season | Buluqute (stream) | N-7.955925, E-36.506322 | 0 | 0 |
| 19/6/2019 | Rainy season | Gembe(stream) | N-7.846611,E-36.661874 | 3 | 0 |

| | | | | | |
|---|---|---|---|---|---|
| 19/6/2019 | Rainy season | Jawe (river) | N-7.957959, E-36.509508 | 0 | 0 |
| 19/6/2019 | Rainy season | Naggo Hagayyo (Swamp) | N-7.954103, E-36.510323 | 0 | 0 |
| 19/6/2019 | Rainy season | Qarqir(stream) | N-7.93517, E-36.508335 | 0 | 0 |
| 19/6/2019 | Rainy season | Semma (river) | N-7.91972, E-36.513993 | 0 | 0 |
| 19/6/2019 | Rainy season | Yachi (Irrigation furrow) | N-7.942895, E-36.512464 | 0 | 0 |
| 19/6/2019 | Rainy season | Burqa(spring) | N-7.811247, E-36.683232 | 0 | 0 |
| 1/8/2019 | Rainy season | Yamo(dam) | N-7.940623, E-36.497171 | 0 | 0 |
| 1/8/2019 | Rainy season | Yisa (stream) | N-7.943158, E-36.506631 | 25 | 0 |
